# A genotype-phenotype study of hereditary multiple exostoses in forty-six Chinese patients

**DOI:** 10.1186/s12881-017-0488-2

**Published:** 2017-11-10

**Authors:** Yuchan Li, Jian Wang, Zhigang Wang, Jingyan Tang, Tingting Yu

**Affiliations:** 1 Department of Pediatric Orthopedics, Shanghai Children’s Medical Center, Shanghai Jiaotong University School of Medicine, Shanghai, China; 2 Institute of Pediatric Translational Medicine, Shanghai Children’s Medical Center, Shanghai Jiaotong University School of Medicine, Shanghai, China; 3 Department of Laboratory Medicine, Shanghai Children’s Medical Center, Shanghai Jiaotong University School of Medicine, Shanghai, China; 4 Department of Hematology and Oncology, Shanghai Children’s Medical Center, Shanghai Jiaotong University School of Medicine, 1678 Dongfang Road, Shanghai, 200127 People’s Republic of China

**Keywords:** Hereditary multiple exostoses, Genotype-phenotype, *EXT* mutations

## Abstract

**Background:**

Hereditary multiple exostoses (HME) is a rare autosomal dominant skeletal disorder that can cause a variety of clinical manifestations. We aimed to evaluate the general clinical phenotypic severity of HME by using a scoring system and correlate the genotypes with different clinical phenotypes in Chinese patients.

**Methods:**

Forty-six patients from different families were prospectively enrolled. The mutations were identified by direct sequencing of PCR-amplified genomic DNA or by multiplex ligation-dependent probe amplification (MLPA). Patients’ demographic data, height, age of onset, number of anatomical sites, forearm deformity, and lower extremity alignment were analysed according to genotype and gender. A scoring system was used to assess the severity of the clinical phenotype.

**Results:**

Thirty (60%) patients presented mutations in the *EXT1* gene, and 16 (32%) presented mutations in the *EXT2* gene. The mean age of onset was 2.96 years. The mean number of involved anatomic sites was 15.35. Male patients had more lesion sites than female patients (15.97 vs. 13.77, *p* = 0.046). The height evaluation illustrated that 67% of the patients (31 of 46) were below the 50th percentile, and the patients with *EXT1* mutations were shorter than those with *EXT2* mutations (*p* = 0.005). Forearm deformity showed a significant correlation with the number of involved anatomical sites (*r* = 0.382, *p* = 0.009). Moreover, a higher total score was found in patients with *EXT1* mutations (*p* = 0.001).

**Conclusions:**

The clinical manifestations of 46 Chinese HME patients were similar to those in previous reports of Western populations. Patients with *EXT1* mutations have a more severe clinical phenotype than patients with *EXT2* mutations.

## Background

Hereditary multiple exostoses (HME) is a rare autosomal dominant condition that is characterized by multiple benign cartilage-capped tumours, primarily at the juxta-epiphyseal region of long bones. The clinical manifestations of HME include short stature, limb length discrepancies, forearm deformities and valgus deformities of the knee and ankle [1]. Two genes, *EXT1* and *EXT2*, located at 8q24.11 and 11p11.2, respectively, have been identified as causing HME [2–4]. These two genes may play different roles in heparan sulfate (HS) biosynthesis.

Several English language publications on genotype–phenotype correlation have indicated that patients with *EXT1* mutations have more severe clinical manifestations [5–7]. However, there are few reports about clinical manifestations of HME in mainland Chinese patients. In this study, we describe the clinical features of HME patients according to genotype and gender and analyse the severity of clinical phenotypes by using a scoring system.

## Methods

From 2013 to 2015, a prospective database was established for patients with multiple exostoses. According to this protocol, patients were initially diagnosed on the basis of radiology. Subsequently, a geneticist performed a genetic analysis of the patients. All patients’ parents and guardians gave written informed consent, and the ethics committee of our hospital approved the study protocols.

### Clinical phenotype study

Patients’ characteristics, including sex, age of onset, and height, were recorded. Because deformities of the spine, hands, and feet were not obvious, the radiological assessment consisted of only a bilateral upper-extremity anteroposterior view with the elbow extended and the forearm supinated, a lateral view of the elbow flexed at 90°, and long standing anteroposterior radiographs of the lower extremities, including the pelvis. On these plain films, exostoses at the proximal and distal ends of the long bones were recorded at 24 anatomic locations (12 on each side of the body). Stature percentiles were determined according to national growth charts [8].

**Fig. 1:**
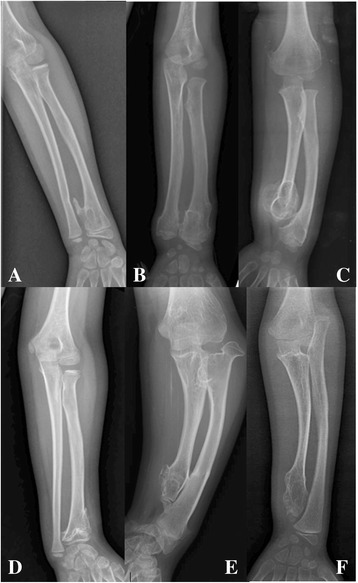
**a**-**b** Grade 2, exostoses of the distal radius or ulna without obvious shortening of either bone or proportionate shortening; **c** Grade 3, Masada type I, exostoses of the distal ulna or radius resulting in a relatively shortened ulna and a bowing radius; **d** Grade 3, Masada type III, exostoses affected distal radius and resulted in a relatively shortened radius; **e** Grade 4, exostoses at the distal ulna and the proximal metaphysis of the radius, a relatively shortened ulna and dislocated radial head described as Masada type IIa; **f** Grade 4, Masada type IIb, the radial head is dislocated without a proximal radial exostosis

Forearm deformities and lower-extremity malalignments were the most common and obvious deformities of appearance. When assessing forearm deformities, we noted that forearms with exostoses at both the distal radius and ulna without obvious deformities or proportional shortening of the radius and ulna cannot be classified according to the Masada classification [9]. Therefore, we defined these deformities as type 0 and established the following ordinal grading system for forearm deformities: Grade 1, no exostoses of the distal forearm; Grade 2, exostoses of the distal radius or ulna without shortening of either bone or proportionate shortening; Grade 3, exostoses of the distal ulna or radius resulting in a relatively shortened ulna and a bowing radius or a relatively shortened radius, including Masada type I and type III; and Grade 4, exostoses of the distal ulna, a relatively shortened ulna, and a dislocated radial head, described as Masada type IIa and type IIb (Fig. 1). On standing plain films, the normal coronal plane alignment of the lower extremities is determined by the mechanical axis passing from the centre of the femoral head to the ankle; mechanical axis deviation (knee varus or valgus) occurs if the axis is not past the centre of the knee joint.

A scoring system was used to assess the severity of the clinical phenotype (Table 1). The scores for the age of onset, height, number of anatomical sites, forearm deformity, and lower extremity alignment were added up to produce the total score. The higher the total score was, the more severe were the clinical manifestations.

**Table 1:** Scoring system used in this study

Parameters			Score
Age of onset (years)			
		>3^a^	1
		<=3	2
Height stature			
		> = P50	0
		<P50	1
		<P25	2
		<P10	3
		<P3	4
Number of involved anatomical sites^b^			
		<15	1
		> = 16	2
Forearm deformity			
	I	No exostosis involvement of the distal forearm	0
	II	With exostosis involvement of the distal radius or ulna without shortening of either bone or proportionate shortening	1
	III	With exostosis involvement of the distal ulna or radius results in relative shortening of the ulna and a bowed radius or relative shortening of the radius, include Masada type I and type III	2
	IV	With exostoses in the distal ulna, a relatively shorter ulna and dislocated radial head described as Masada type IIa and IIb	3
Alignment of low extremity			
		Normal	0
		Valgus/Varus	1

^a^HME has penetrance of 50% by the age of three and a half [8]; in this study less than three years was defined as 1 point

^b^The mean number of involved anatomical sites in this study was 15

### Genetic study

Genomic DNA was extracted from patient peripheral blood samples using a QIAamp Blood DNA Mini kit® (Qiagen GMBH, Hilden, Germany). Primers for the amplification of the *EXT1* and *EXT2* genes (GenBank accession numbers NM_000127.2 and NM_207122.1, respectively) were designed using Primer3 online software (primer sequences are available upon request). All exons and exon–intron boundaries of each gene were amplified by polymerase chain reaction (PCR) (Takara Bio, Dalian, China). The amplified products were purified from an agarose gel using a QIAquick Gel Extraction Kit (Qiagen GMBH) and sequenced with an ABI3730XL sequencer (Applied Biosystems, Foster City, CA, USA). For PCR-negative patients, multiplex ligation-dependent probe amplification (MLPA) analysis was performed using the SALSA MLPA probe mix P215-B2 EXT kit (MRC Holland, Amsterdam, Netherlands) according to the manufacturer’s protocol. Data analysis and interpretation were performed using GeneMarker® software (Softgenetics, State College, PA, USA).

### Statistical methods

The statistical analysis was performed using Statistical Package for the Social Sciences version 17.0. Descriptive statistics were described as the means ± SD and ranges (minimum to maximum). Categorical data were analysed with Pearson’s chi-squared test or Student’s *t*-test. For nonparametric data, the Mann–Whitney U test was performed to check for significant differences between groups. A Spearman’s rho or Kendall’s tau correlation analysis was used to assess relationships between ordinal variables. Differences were considered significant at a *p*-value of <0.05.

## Results

### General clinical aspects

Patients who were unwilling to undergo further X-rays were excluded from the study. Fifty subjects from different families were prospectively enrolled, including 30 (60%) with *EXT1* mutations and 16 (32%) with *EXT2* mutations (Table 2); four patients (8%) with no identifiable mutations were excluded from the study. Thus, the study cohort consisted of 46 patients, including 33 males (*EXT1*/*EXT21*, 22/11) and 13 females (*EXT1*/*EXT2*, 8/5) (Table 3), with a mean age of 9.3 years (range 5.3–15.6 years, SD = 3.06). The mean age of onset was 2.96 years (range 1–12.5 years, SD = 2.237). The mean number of involved anatomical sites was 15.35 (range 7–20, SD = 3.38).

**Table 2:** *EXT1* and *EXT2* mutations in patients

Case Number	Mutant gene		Variant type
	EXT1	EXT2	
1	c.1567delC		Small deletion(frameshift)
2	c.1722 + 2 T > G, I8 + 2 T > G		Splice site
3	c.651-664delinsTTT		Frameshift
4	c.680delG		Small deletion(frameshift)
5		c.1016G > A	Missense
6		c.544C > T	Nonsense
7	c.1879_1881delCAC		Small deletion(codon)
8	c.1108G > T		Nonsense
9	c.335delA		Small deletion(frameshift)
10		EXT2 ALL EXON del	
11		c.IVS4 + 1G > T	Splice site
12		c.1075A > T	Nonsense
13	c.942_943delAG		Small deletion(frameshift)
14	c.1784_1785delGC		Small deletion(frameshift)
15		c.IVS2 + 2_5delTAGG	Splice site
16	c.1469delT		Small deletion(frameshift)
17		c.67C > T	Nonsense
18		c.678C > A	Nonsense
19	c.ins247C		Small insert(frameshift)
20		c.1188G > A	Nonsense
21		c.924C > A	Nonsense
22		c.627-2_630delinsT	Splice site
23	c.1705delG		Small deletion(frameshift)
24	c.1551G > A		Nonsense
25	c.1019G > A		Missense
26	c.1930A > T		Missense
27	c.1165-1G > T		Splice site
28	c.1469delT		Small deletion(frameshift)
29		c.925_928dupCCAC	Small duplication(frameshift)
30	c.635delG		Small deletion(frameshift)
31	c.1883 + 1G > A(het)		Splice site
32		c.382C > T	Missense
33	EXT1 E4 del		
34		c.1182delG	Small deletion(frameshift)
35		c.910C > T	Nonsense mutation
36	EXT1 E2-E11 del		
37	c.247dupC		Small duplication(frameshift)
38	EXT1 E4 del		
39	c.1930A > T		Missense
40	c.200C > A;		Missense
41		c.1181G > A	Nonsense
42	EXT1 ALL EXON del		
43	EXT1 ALL EXON del		
44	c.659G > A		Missense
45	c.354dupA		Small duplication(frameshift)
46	c.1911C > A		Nonsense

**Table 3:** The different *EXT* mutations according to gender

Gender	EXT1	EXT2
Male	22	11
Female	8	5

*p* = 1.000, Chi-squared

The height evaluation illustrated that 67% of the patients (31 of 46) were below the 50th percentile. Knee valgus deformity was observed in 41 limbs. No varus deformity was observed in this study. Forearm deformities are more common, and only seven forearms were not involved (grade I), 36 forearms were grade II, 34 forearms were grade III, and 15 forearms were grade IV. There is no correlation between forearm deformity and stature (*p* = 0.499). However, forearm deformity had a significant correlation with the number of involved anatomical sites (*r* = 0.382, *p* = 0.009, Spearman’s rho) at a 0.05 level. Moreover, a strong relationship with lower limb malalignment (*r* = 0.885, *p* < 0.001, Kendall tau b test) was observed in our data. A comparison of deformities of the left forearm to those of the right side revealed no significant difference in the body side distribution of forearm deformity (*p* = 0.987, Mann–Whitney test).

### Gene mutation-related analysis

**Fig. 2:**
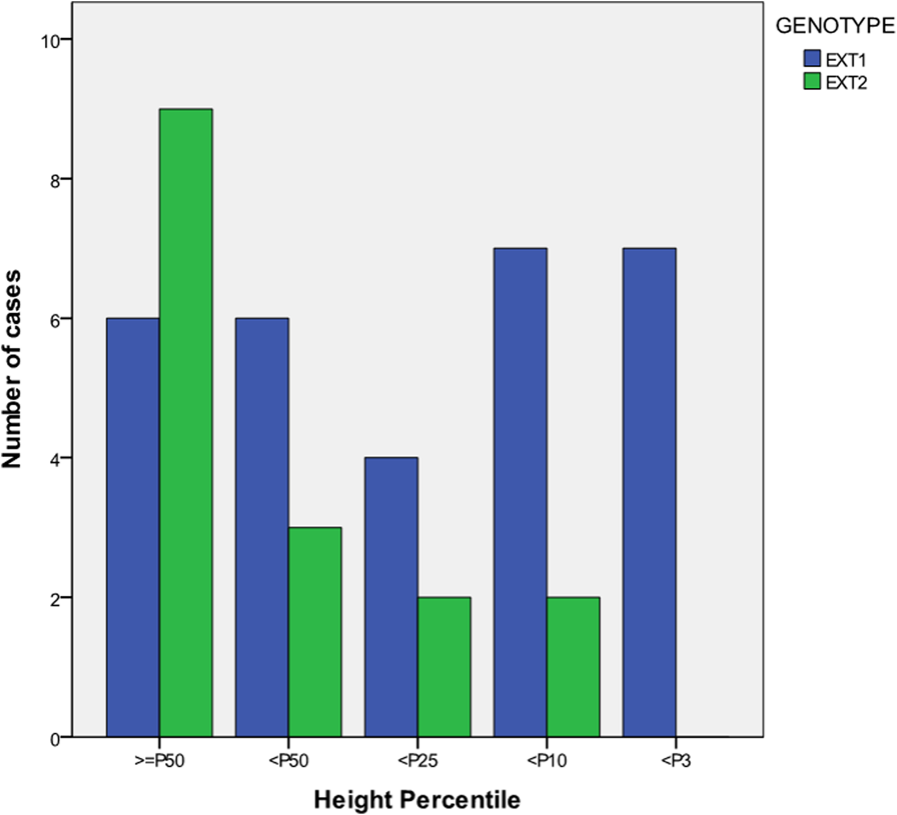
Distribution of height percentile in *EXT1* and *EXT2* genotypes. The bar indicates the number of patients. Compared with *EXT1* mutation patients, there were 9 of 16 patients (56.25%) with EXT2 mutations whose height was greater than the 50th percentile, whereas no patients’ height fell below the 3rd percentile. (*p* = 0.005, Mann–Whitney test)

**Fig. 3:**
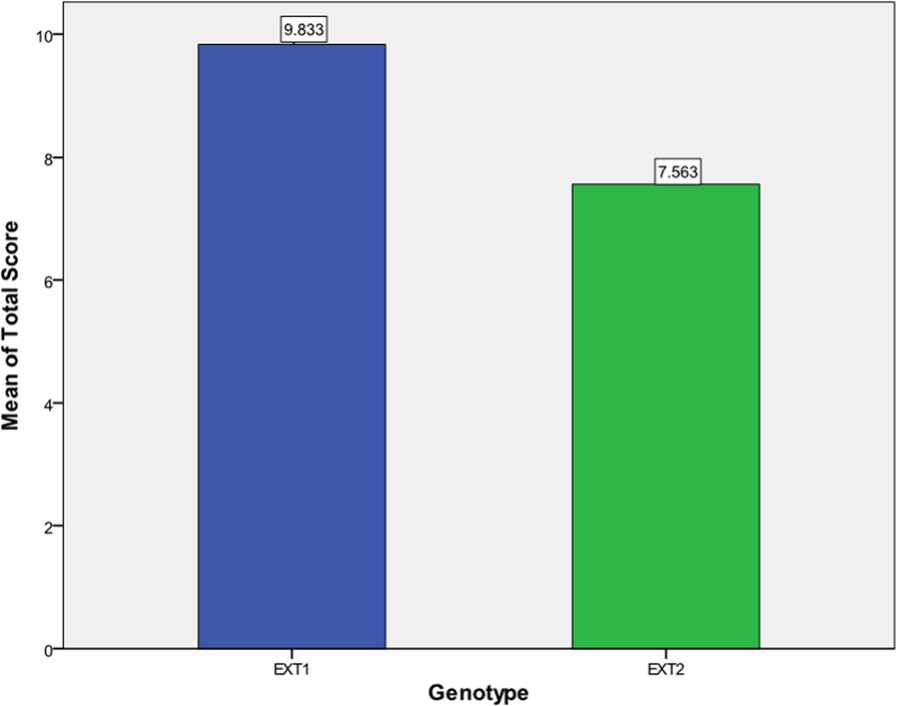
Total score of different *EXT* genotypes. The mean total score of patients with *EXT1* mutations was 9.83, higher than that of patients with *EXT2* mutations. (*p* = 0.001, *t-*test)

We found no significant differences in age (*p* = 0.658) or age of onset (*p* = 0.957) with regard to the *EXT* mutation. There was also no significant difference between the genotypes in gender distribution (*p* = 1.000, chi-squared). The *EXT1* patients were shorter than the *EXT2* patients (*p* = 0.005, Mann–Whitney test) (Fig. 2); 46% (14 of 30) of the patients with *EXT1* mutations were below the 10th percentile compared with 12.5% (2 of 16) of the patients with *EXT2* mutations. The mean number of involved anatomical sites was slightly greater in patients with *EXT1* mutations compared with those with *EXT2* mutations, but this difference was not significant (*p* = 0.110, *t-*test). We also found no significant differences between the two genotypes in forearm deformities or lower extremity malalignment (*p* = 0.432 and *p* = 0.403, respectively) (Table 4). However, we observed a higher total score in the patients with *EXT1* mutations (*p* = 0.001, *t-*test) (Fig. 3).

**Table 4:** Comparison of clinical features for patients according to genotype and gender

P-value	Genotype	Gender
Height	0.005	0.754
Forearm deformity	0.432	0.413
Lower limb malalignment	0.403	0.855
Age of onset	0.957	0.915
Number of involved anatomical sites	0.110	0.046
Total score	0.001	0.513

### Gender-related analysis

Male patients had more lesion sites than female patients (15.97 vs. 13.77), (*p* = 0.046, *t-*test); however, we found no significant differences in other parameters between male and female patients (Table 4).

### Age-related analysis

**Fig. 4:**
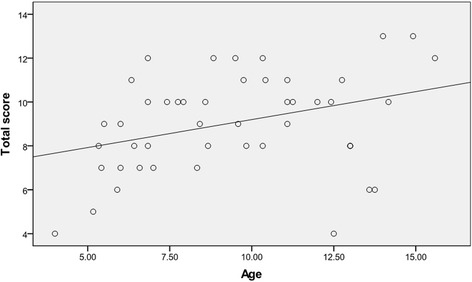
A linear correlation between age and total score: there was a trend towards increased total score in older children. Older children had a higher total score, indicating that the severity of the clinical phenotype was age-dependent

By contrast, with gender, we observed that several parameters showed progressive tendencies with increasing age, including the number of involved anatomical sites (*r* = 0.439, *p* = 0.002), forearm deformities (*r* = 0.316, *p* = 0.032), and lower extremity alignments (*r* = 0.438, p = 0.002). Similarly, we also found a linear correlation between age and total score (*r* = 0.346, *p* = 0.018), as shown in Fig. 4.

## Discussion

The prevalence of HME in Western populations is estimated at 1:50,000 [10], whereas it is uncertain in mainland China. Only one publication on the clinical manifestations of Chinese HME patients was found when searching the PubMed databases, and it was based on a literature review. Most publications in Chinese are case reports and mutation screenings [11]. Our study is the first prospective genotype-phenotype analysis of HME in Chinese patients.

Hereditary multiple exostoses (HME) is one of the most common benign musculoskeletal disorders. The majority of HME cases are caused by loss-of-function mutations in *EXT1* or *EXT2*, and linkage occurs in approximately one-half and one-third of HME families, respectively. Mutations in *EXT1* and *EXT2* were responsible for 60 and 32% of the cases in this study, respectively. These mutation frequencies are similar to those in Western populations but are quite different from those in a mainland China population in a previous study (14% *EXT1* and 33% *EXT2*) [12]. These findings suggest that regional differences in *EXT1* and *EXT2* mutation frequencies occur in China. Moreover, the use of MLPA has dramatically increased the detection of mutations: it can identify large-fragment deletions and duplications based on hybridization and ligation, followed by the amplification of the ligation products.

The *EXT* genes encode homologous Golgi-associated glycosyltransferases that are involved in the chain elongation step of HS biosynthesis. Studies have indicated that HS influences various important processes in skeletogenesis and skeletal growth and morphogenesis. Loss-of-function mutations in the *EXT1* and *EXT2* genes lead to HS deficiency [13, 14]. *EXT2* forms a complex with *EXT1*, and this complex is believed to be the functional HS polymerase. However, the *EXT1* and *EXT2* proteins have different roles: *EXT1* exerts a predominantly biological function, whereas *EXT2* is speculated to assist in the folding and transport of *EXT1* to the Golgi complex instead of elongation of the HS backbone [15]. Therefore, the variable clinical phenotypes of HME might result from the different gene mutations.

Many researchers have reported the genotype–phenotype correlation in HME [5, 7, 16, 17]. Francannet et al. divided their subjects into severe or moderate groups by using five factors, and they found that most severe forms of the disease and malignant transformation were associated with *EXT1* mutations [5]. Pedrini et al. classified their patients based on the presence of deformities and functional limitations and reported that being male, having more than 20 affected skeletal sites, and having an *EXT1* mutation were each correlated with increased risk of a severe phenotype [17].

In this study, we observed that 67% of patients were below the 50th percentile, confirming reports that patients with HME have a shorter stature and that patients with *EXT1* mutations are shorter than patients with *EXT2* mutations. However, we found no significant difference in the number of involved anatomical sites between genotypes, and these data are in contrast to those of previous studies [5–7, 17]. An explanation for this finding may be that we did not evaluate exostosis involvement of flat bones, whereas some authors have reported a higher degree of anatomical burden and more flat bone involvement in *EXT1* patients than in *EXT2* patients [7, 16]. Thus, we speculate that the different functions of the *EXT* genes in HS biosynthesis may lead to different exostosis involvement of the flat bones.

The forearm deformities classified by Masada are a characteristic trait of patients with HME. The prevalence of forearm deformities is as high as 30–60% [9, 10]. Our data are in accord with this prevalence; we found that 53% (49 of 92) of forearms showed obvious deformities. Taniguchi et al. [18] found that increasing forearm involvement was associated with HME diagnosis at an earlier age, a greater number of generalized exostoses, a shorter stature, a greater number of exostoses affecting the knee, and increased valgus deformity of the ankle. By contrast, we found no correlations between the grade of forearm deformity and the age of onset or stature. However, strong associations with the number of involved anatomical sites and lower limb malalignment were observed; the more severe the forearm deformity, the higher the number of anatomical sites involved and the greater the lower limb malalignment. Meanwhile, we found no predominant side when comparing the forearm deformities; the degree of forearm deformity on both sides was similar, which is in contrast to Jäger et al. who reported more exostoses affecting the right side [19].

In this study, we used a scoring system to assess the severity of the clinical phenotype. Forearm deformity and lower extremity malalignment were selected to represent limb deformities, and the age of onset, stature, and number of involved anatomical sites represented the general aspect of exostoses. After the grade of the deformities was defined, it was easier to evaluate the severity of the clinical phenotype according to the total score: the higher the score, the more severe the phenotype. Although there were no significant differences between the two genotypes regarding these parameters except for height, the total score of patients with *EXT1* mutations was higher than that of patients with *EXT2* mutations. This result is consistent with previous investigations indicating the more severe clinical phenotype of *EXT1* mutations.

Some authors have reported sex-dependent differences in HME; the prevalence seems to be higher in males [20], and male patients usually present with more exostoses and shorter stature than females [7, 17, 19]. Our data corroborate these reports; 72% (33 of 46) of our patients were male, and we observed more lesion sites in male patients. However, there were no gender-related differences in any other parameters.

Our results demonstrated the age-related phenotypic penetrance of HME. As the exostoses continue to enlarge until epiphyseal fusion occurs, the number of involved anatomical sites increased with age; forearm deformities and lower extremity malalignments became more severe in older children; and the total score showed a strong association with age (Fig. 4). We did not observe the most severe complication, malignant transformation, in this study because this complication is also age-related; it is usually diagnosed at an average age of 31 years and seldom occurs in the first decade of life [1, 21]. However, Schmale et al. [22] reported two cases of skeletally immature patients who presented with malignant transformation. Therefore, adolescent patients should be followed carefully to detect signs of early malignant transformation.

We note several limitations of this study. First, this was a cross-sectional study; as most clinical manifestations are age-related, a longitudinal observational study would decrease the risk of confounding factors. Second, the sample size limitation may have led to some bias. Third, the study was based on radiographic measurements and did not include an evaluation of functional impairment. Finally, the scoring system used in this study may be arbitrary; however, there are still no universally accepted criteria to categorize phenotype severity, although several methods have been mentioned to grade severity. The five factors used to evaluate severity in this study are characteristic traits of HME, but they need to be further verified by statistics.

## Conclusions

In this study, we have confirmed that several clinical features of HME in Chinese patients are similar to those in most Western populations. Patients with HME are frequently of short stature; in this study, patients with *EXT1* mutations were shorter in stature compared with *EXT2* patients, but we did not observe any significant differences between other parameters including age of onset, number of involved anatomical sites, forearm deformities and lower limb malalignment. However, it would seem that patients with *EXT1* mutations have a more severe clinical phenotype compared with those with *EXT2* mutations, which we demonstrated in terms of the total score by our scoring system. Although this study has some limitations, it may be helpful for further research on HME.

## Abbreviations

HME: Hereditary multiple exostoses
HS: Heparan sulfate
MLPA: Multiplex ligation-dependent probe amplification
PCR: Polymerase chain reaction

## References

[1] Stieber JR, Dormans JP (2005). Manifestations of hereditary multiple exostoses. J Am Acad Orthop Surg.

[2] Ahn J, Lüdecke HJ, Lindow S (1995). Cloning of the putative tumour suppressor gene for hereditary multiple exostoses (EXT1). Nat Genet.

[3] Stickens D, Clines G, Burbee D (1996). The EXT2 multiple exostoses gene defines a family of putative tumour suppressor genes. Nat Genet.

[4] Wuyts W, Van Hul W, Wauters J (1996). Positional cloning of a gene involved in hereditary multiple exostoses. Hum Mol Genet.

[5] Francannet C, Cohen-Tanugi A, Le Merrer M (2001). Genotype-phenotype correlation in hereditary multiple exostoses. J Med Genet.

[6] Porter DE, Lonie L, Fraser M (2004). Severity of disease and risk of malignant change in hereditary multiple exostoses: a genotype-phenotype study. J Bone Joint Surg Br..

[7] Clement ND, Porter DE (2014). Hereditary multiple exostoses: anatomical distribution and burden of exostoses is dependent upon genotype and gender. Scott Med J.

[8] Li H, Ji CY, Zong XN (2009). Height and weight standardized growth charts for Chinese children and adolescents aged 0 to 18 years. Chin J Pediatr.

[9] Masada K, Tsuyuguchi Y, Kawai H (1989). Operations for forearm deformity caused by multiple osteochondromas. J Bone Joint Surg Br.

[10] Schmale GA, Conrad EU, Raskind WH (1994). The natural history of hereditary multiple exostoses. J Bone Joint Surg Am.

[11] Guo XL, Deng Y, Liu HG (2014). Clinical characteristics of hereditary multiple exostoses: a retrospective study of mainland chinese cases in recent 23 years. J Huazhong Univ Sci Technolog Med Sci.

[12] Xu L, Xia JH, Jiang HJ (1999). Mutation analysis of hereditary multiple exostoses in the Chinese. Hum Genet.

[13] Huegel J, Sgariglia F, Enomoto-Iwamoto M (2013). Heparan sulfate in skeletal development, growth, and pathology: the case of hereditary multiple exostoses. Dev Dyn.

[14] Busse-Wicher M, Wicher KB, Kusche-Gullberg M (2014). The extostosin family: proteins with many functions. Matrix Biol.

[15] Busse M, Feta A, Presto J (2007). Contribution of EXT1, EXT2, and EXTL3 to heparan sulfate chain elongation. J Biol Chem.

[16] Alvarez CM, De Vera MA, Heslip TR (2007). Evaluation of the anatomic burden of patients with hereditary multiple exostoses. Clin Orthop Relat Res.

[17] Pedrini E, Jennes I, Tremosini M (2011). Genotype-phenotype correlation study in 529 patients with multiple hereditary exostoses: identification of “protective” and “risk” factors. J Bone Joint Surg Am.

[18] Taniguchi K (1995). A practical classification system for multiple cartilaginous exostosis in children. J Ped Orthop.

[19] Jäger M, Westhoff B, Portier S, Leube B (2007). Clinical outcome and genotype in patients with hereditary multiple exostoses. J Othop Res.

[20] Legeai-Mallet L, Munnich A, Maroteaux P, Le Merrer M (1997). Incomplete penetrance and expressivity skewing in hereditary multiple exostoses. Clin Genet.

[21] Pierz KA, Stieber JR, Kusumi K (2002). Hereditary multiple exostoses: one center’s experience and review of etiology. Clin Orthop.

[22] Schmale GA, Hawkins DS, Rutledge J (2010). Malignant progression in two children with multiple osteochondromas. Sarcoma.

